# Mental Illnesses-Associated Fxr1 and Its Negative Regulator Gsk3β Are Modulators of Anxiety and Glutamatergic Neurotransmission

**DOI:** 10.3389/fnmol.2018.00119

**Published:** 2018-04-12

**Authors:** Jivan Khlghatyan, Alesya Evstratova, Simon Chamberland, Aleksandra Marakhovskaia, Arash Bahremand, Katalin Toth, Jean-Martin Beaulieu

**Affiliations:** ^1^Department of Pharmacology and Toxicology, University of Toronto, Toronto, ON, Canada; ^2^Department of Psychiatry and Neuroscience, Faculty of Medicine, Université Laval, Québec City, QC, Canada

**Keywords:** fragile X proteins, GSK-3, mood disorders, CRISPR/Cas9, frontal cortex, AMPAR

## Abstract

Genetic variants of the fragile X mental retardation syndrome-related protein 1 (*FXR1)* have been associated to mood regulation, schizophrenia, and bipolar disorders. Nonetheless, genetic association does not indicate a functional link of a given gene to neuronal activity and associated behaviors. In addition, interaction between multiple genes is often needed to sculpt complex traits such as behavior. Thus, modulation of neuronal functions by a given gene product, such as Fxr1, has to be thoroughly studied in the context of its interactions with other gene products. Glycogen synthase kinase-3 beta (GSK3β) is a shared target of several psychoactive drugs. In addition, interaction between functional polymorphisms of *GSK3b* and *FXR1* has been implicated in mood regulation in healthy subjects and bipolar patients. However, the mechanistic underpinnings of this interaction remain unknown. We used somatic CRISPR/Cas9 mediated knockout and overexpression to investigate the impact of Fxr1 and its regulator Gsk3β on neuronal functions directly in the adult mouse brain. Suppression of Gsk3β or increase of Fxr1 expression in medial prefrontal cortex neurons leads to anxiolytic-like responses associated with a decrease in AMPA mediated excitatory postsynaptic currents. Furthermore, Fxr1 and Gsk3β modulate glutamatergic neurotransmission via regulation of AMPA receptor subunits GluA1 and GluA2 as well as vesicular glutamate transporter VGlut1. These results underscore a potential mechanism underlying the action of Fxr1 on neuronal activity and behaviors. Association between the Gsk3β-Fxr1 pathway and glutamatergic signaling also suggests how it may contribute to emotional regulation in response to mood stabilizers, or in illnesses like mood disorders and schizophrenia.

## Introduction

The fragile X mental retardation syndrome-related protein 1 (Fxr1) is a member of a small family of RNA binding proteins that also comprises the fragile X mental retardation protein 1 (Fmr1) and Fxr2 (Siomi et al., 1995). FXR family proteins are enriched in the brain with Fxr1 being expressed in neurons, astrocytes, oligodendrocytes, microglia, and endothelial cells of mouse cortex (Tamanini et al., 1997; Bakker et al., 2000; Cook et al., 2011; Thomsen et al., 2013; Zhang et al., 2014). The neuronal functions of this family have mostly been studied in the context of fragile X syndrome and autism spectrum disorders (Bardoni et al., 2001; Pfeiffer and Huber, 2009). However, genome-wide association studies (GWAS) have linked *FXR1* to schizophrenia and bipolar disorders (Consortium, 2014; Hauberg et al., 2016; Liu et al., 2016; Takata et al., 2017), therefore indicating its possible wider roles in mental illnesses. Nonetheless, genetic association does not always indicate a direct mechanistic link to neuronal activity and associated behavior (Boyle et al., 2017). Moreover, complex traits are often influenced by interactions between multiple genes.

We identified genetic polymorphisms in *FXR1* and *GSK3B* that are linked to differential expression of their respective mRNAs in the human dorsolateral prefrontal cortex (DLPFC) (Del’Guidice et al., 2015). Interaction between these polymorphisms contributes to mood regulation in healthy subjects in whom higher *FXR1* expression is associated to greater emotional stability, except in the context of higher *GSK3B* expression (Del’Guidice et al., 2015). Furthermore, an interaction between these genetic variants has also been linked to symptom severity in bipolar patients (Bureau et al., 2017). The *GSK3B* gene encodes glycogen synthase kinase-3 beta (Gsk3β), a serine-threonine kinase. Inhibition of Gsk3β is a consequence of treatment with several psychoactive drugs including antipsychotics, antidepressants, ketamine and mood stabilizers (Beaulieu et al., 2009; Beurel et al., 2011). Fxr1 is directly phosphorylated by Gsk3β and negatively regulated by this kinase (Del’Guidice et al., 2015). Conversely, chronic treatment with the mood stabilizers —lithium, lamotrigine or valproate— or other manipulations leading to an inhibition of Gsk3β, elevate Fxr1 levels (Del’Guidice et al., 2015).

Mental illnesses are believed to be associated to a misregulation of the neuronal excitation/inhibition balance (Nelson and Valakh, 2015; Foss-Feig et al., 2017; Lener et al., 2017). Ionotropic glutamate receptors, α-Amino-3-hydroxy-5-methyl-4-isoxazole Propionic-Acid (AMPA) and *N*-methyl-D-aspartate (NMDA) are the major mediators of excitatory transmission in the brain. Changes in AMPA or NMDA receptors could be one of the causes of imbalance of neuronal activity. Moreover, alterations in glutamatergic neurotransmission have been widely implicated in mental illnesses (Javitt, 2004; Lener et al., 2017). Thus, mechanistic contribution of genetic risk factors for schizophrenia and mood disorders to the regulation of glutamatergic neurotransmission in the nervous system is of a particular interest.

We used CRISPR/Cas9 mediated somatic gene knockout (sKO) in combination with adeno-associated viral vector (AAV) driven gene overexpression to investigate the consequences of altered Fxr1 and Gsk3β expression in the adult medial prefrontal cortex (mPFC) on neuronal activity and associated behaviors. Augmentation of Fxr1 and reduction of Gsk3β expression resulted in anxiolytic-like behaviors and decrease in AMPA mediated spontaneous excitatory currents. Further investigation of underlying mechanism revealed that increase in Fxr1 and decrease in Gsk3β expression leads to AMPA receptor composition change most likely due to alteration of trafficking of both synaptic GluA1 and GluA2 subunits. Changes in AMPA receptor subunits were accompanied with a decrease in vesicular glutamate transporter 1 (Vglut1) indicating pre- and post-synaptic changes of glutamatergic neurotransmission. Overall, our results uncovered an implication of Fxr1 and its regulator Gsk3β in the control of synaptic components of glutamatergic neurotransmission. These results underscore a mechanism by which Fxr1 contributes to the regulation of neuronal activity and suggest how it could be implicated in emotional regulation.

## Materials and Methods

### Experimental Animals

All experiments conducted in this study were approved by either the Université Laval or University of Toronto Institutional Animal Care Committee in line with guidelines from the Canadian Council on Animal Care. For all the experiments C57BL/6J male (Jackson Laboratory, Bar Harbor, ME, United States) mice were used. Littermates were housed 3–4 per cage in a humidity-controlled room at 23°C on a 12 h light dark cycle with *ad libitum* access to standard mouse chow and water. At the time of experiment, mice were 3–4 months old and weighed approximately 25–30 g. Animals were all drug naïve and were used only for single experiments.

### DNA Constructs

**FIGURE 1:**
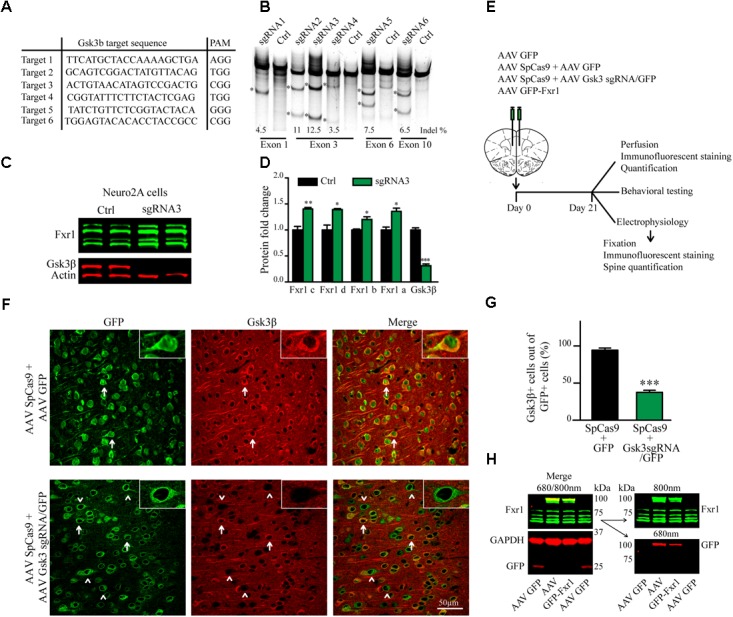
CRISPR/Cas9 mediated somatic knockout (sKO) of *Gsk3b* in medial prefrontal cortex (mPFC). **(A)** *Gsk3b* targeting sequences and corresponding protospacer adjacent motifs (PAMs). **(B)** Evaluation of *Gsk3b* targeting sgRNAs by SURVEYOR assay 2 days after transfection of sgRNAs and SpCas9. **(C)** Western blot analysis of Gsk3β and Fxr1 expression in Neuro2A cells 7 days after transfection of CRISPR/Cas9 constructs (Fxr1 bands from top to bottom: isoform c, isoform d, isoform b, isoform a). **(D)** Quantification of Gsk3β and Fxr1 expression levels after CRISPR/Cas9 mediated knockout (Fxr1 isoform c Ctrl 1 ± 0.07, Gsk3 KO 1.4 ± 0.028; Fxr1 isoform d Ctrl 1 ± 0.09, Gsk3 KO 1.39 ± 0.02; Fxr1 isoform b Ctrl 1 ± 0.018, Gsk3 KO 1.2 ± 0.05; Fxr1 isoform a Ctrl 1 ± 0.05, Gsk3 KO 1.35 ± 0.06; Gsk3β Ctrl 1 ± 0.04, Gsk3 KO 0.31 ± 0.03, ^∗^*p* < 0.05, ^∗∗^*p* < 0.01, ^∗∗∗^*p* < 0.001, one way ANOVA). **(E)** Schematic diagram of experimental design. **(F)** Immunostaining of virus injected brain sections with Gsk3β antibody (Gsk3β red, GFP green). Arrows indicate GFP + Gsk3β+ (doublepositive) cells, arrowheads indicate cells only positive for GFP. **(G)** Quantification of Gsk3β+ cells in the population of GFP+ virus infected cells (Ctrl 94.35% ± 2.83 256cells, Gsk3sgRNA 37.51% ± 2.80 293cells, *n* = 3 mice, ^∗^*p* < 0.05, ^∗∗^*p* < 0.01, ^∗∗∗^*p* < 0.001, Student’s *t*-test). **(H)** Western blot analysis of GFP-Fxr1 fusion protein expression in mPFC of virus injected mice, GFP-Fxr1 band is detected with both Fxr1 and GFP antibodies (overexpression of GFP-FXR1 was 2.95 ± 0.47 fold over endogenous; *n* = 4, *p* < 0.05, Student *T*-test). Error bars show standard error of the mean (SEM).

To knockout (KO) *Gsk3b* gene 20-nt target sequences in exons of the gene were selected using online CRISPR design tool^1^ to minimize off-target activity. For *in vitro* evaluation of *Gsk3b* KO by SURVEYOR assay (**Figure 1B**), guide oligonucleotides were cloned into pX330 [pX330-U6-Chimeric_BB-CBh-hSpCas9 was a gift from Feng Zhang (Addgene # 42230)] (Cong et al., 2013) all in one vector by single step cloning using BbsI restriction sites (Ran et al., 2013). For *in vitro* evaluation of *Gsk3b* KO by Western blot (**Figures 1C,D**), the most active guide (gRNA3) was cloned into pX459 vector [pSpCas9(BB)-2A-Puro (PX459) V2.0 was a gift from Feng Zhang (Addgene plasmid # 62988)] (Ran et al., 2013). Sequences of all constructs were verified.

To generate sgRNA expressing AAV viral vector (pAAV Gsk3sgRNA/GFP) preparation the most active guide (gRNA3) was cloned into pX552 [pX552 was a gift from Feng Zhang (Addgene plasmid # 60958)] (Swiech et al., 2015) vector by single step cloning using SapI restriction sites. AAV SpCas9 (pX551) was a gift from Feng Zhang (Addgene plasmid # 60957) (Swiech et al., 2015). AAV GFP-Fxr1 (Fxr1 over) neuron-specific AAV vector was described previously (Del’Guidice et al., 2015).

### Cell Line Culture and Transfection

Neuro-2A (N2A) cells were grown in high glucose DMEM containing 10% FBS, penicillin/streptomycin and L-glutamine (HyClone-GE Healthcare, Logan, UT, United States). Cells were maintained at 37°C in 5% CO2 atmosphere and transfected using Lipofectamine 2000 (Thermo Fisher Scientific, Waltham, MA, United States) according to manufacturer’s protocols.

To test the activity of *Gsk3b* sgRNAs by SURVEYOR assay (**Figure 1B**), 50–70% confluent N2A cells were transfected with all in one pX330 based construct (pX330 vectors with guide targeting *Gsk3b*) and lysed 2 days after transfection.

To test the activity of *Gsk3b* sgRNA3 by Western blot and establish regulation of Fxr1 by Gsk3β (**Figures 1C,D**), 50–70% confluent N2A cells were transfected with all in one px459 based constructs (pX459 vectors with guide targeting *Gsk3b*). To select only transfected cells, 48 h after transfection cells were incubated with 3 μM puromycin for 72 h followed by 48 h incubation without puromycin. Cells were washed and lysed on the day 7 after transfection.

### Genomic DNA Extraction and SURVEYOR Assay

For functional testing of sgRNAs, 50–70% confluent N2A cells were transfected with all in one pX330 based constructs (pX330 vectors with guides targeting *Gsk3b*). Cells transfected with pX330 only served as negative control. Cells were lysed 48 h after transfection by tail buffer (Tris pH = 8.0 0.1M, NaCl 0.2M, EDTA 5mM, SDS 0.4% and proteinase K 0.2 mg/ml), and DNA was precipitated using isopropanol followed by centrifugation (13000 g 15 min). DNA was resuspended in TE Buffer (10 mM Tris pH 8.0, 0.1 mM EDTA) and used for downstream analysis. Functional testing of individual sgRNAs was performed by SURVEYOR nuclease assay (Transgenomics, Omaha, NE, United States) using PCR primers listed in **Table 1**. Band intensity quantification was performed as described (Ran et al., 2013).

**Table 1:** PCR primers used in the SURVEYOR assay.

Gene exon	Forward primer sequence	Reverse primer sequence
Gsk3b exon 1	TCTTCCAGGAAAGGGAGGTGA	AGGCACTGGAGCACTTGAAA
Gsk3b exon 3	GGTTCCTCTTGCCCCCTATTA	TTCTCATTGGCATTTCCACGC
Gsk3b exon 6	GCTAACACCTGACACTCACTT	CTGTGAGCACGTCTTTTTGC
Gsk3b exon 10	TAGCAAGCAGTTTGCCCCAC	AGTCCATGATAGTGGAGGGGA

### AAV Viral Particle Preparation

For all viral vector preparations, AAV serotype 5 viral particles were produced by the University of North Carolina (UNC) Vector core facility. AAV GFP (AAV5 hSYN EGFP) and AAV KORD (AAV9-CaMKII-HA-KORD-IRES-mCitrine) (Vardy et al., 2015) were purchased from UNC Vector core facility (Chapel Hill, NC, United States).

### Stereotaxic Injections

Three weeks before the behavioral tests and electrophysiology recordings, bilateral injection of the virus was made in the mPFC. Mice were anesthetized with a preparation of ketamine 10 mg/ml and xylazine 1 mg/ml (0.1ml/10 g, i.p.). The animal was placed in a stereotaxic frame, and the skull surface was exposed. Two holes were drilled at injection sites and 1 μl of virus (AAV GFP-Fxr1 4.4 × 10^12^vg/ml or AAV GFP or AAV KORD 3 × 10^12^vg/ml or 1:1 AAV mixture: AAV SpCas9 2.6 × 10^12^vg/ml and AAV Gsk3sgRNA/GFP 5.4 × 10^12^vg/ml or AAV SpCas9 and AAV GFP 4.5 × 10^12^vg/ml) was injected using nanoliter injector with microsyringe pump controller (WPI) at the speed of 4 nl per second. Following coordinates were used: anterior-posterior (AP), +2.4 mm anterior to bregma; mediolateral (ML), ± 0.5 mm; dorsoventral (DV), 1.7 mm below the surface of the brain. All measures were taken before, during, and after surgery to minimize animal pain and discomfort. These measures included: using local analgesics on the site of incision, using heating pad during surgery and recovery period to keep an optimal body temperature for mice, making minimal incisions on the head skin and minimal size of the hole drilled in the skull, making a proper suture of the skin, so it’s not itchy for mice, using analgesics 24–48 h post surgery.

### Acute Slice Preparation

Mice were anesthetized with isoflurane followed by rapid cervical dislocation. Cortical slices (300 μm) were prepared from mice (3 weeks after injection of viruses) using a vibrating blade microtome (Leica Biosystem, Wetzlar, Germany). Slices were prepared using ice-cold artificial cerebrospinal fluid (ACSF) containing: NaCl 87 mM, NaHCO3 25 mM, KCl 2.5 mM, NaH2PO4 1.25 mM, MgCl2 7 mM, CaCl2 0.5 mM, glucose 25 mM and sucrose 75 mM. Right after sectioning, slices were placed in oxygenated ACSF at 32°C for 30 min, transferred to extracellular ACSF and maintained at room temperature prior to experiments. All recordings were performed with extracellular ACSF containing: NaCl 124 mM, NaHCO3 25 mM, KCl 2.5 mM, MgCl2 1.5 mM, CaCl2 2.5 mM and glucose 10 mM, equilibrated with 95% O2/5% CO2, pH7.4, maintained at 31–33°C and perfused at a rate of 2–3 mL/min.

### Electrophysiology

Whole-cell current-clamp and voltage-clamp recordings were made with glass electrodes (4–6.5 MΩ) filled with a solution containing: K-gluconate 120 mM, KCl 20 mM, MgCl2 2 mM, EGTA 0.6 mM, MgATP 2mM, NaGTP 0.3 mM, Hepes 10 mM, phosphocreatine 7 mM or Cs-gluconate 100 mM, NaCl 8 mM, MgCl2 5 mM, EGTA 0.6 mM, MgATP 2 mM, NaGTP 0.3 mM, Hepes 10 mM, phosphocreatine 7 mM, QX-314 1, spermine 0.1 mM (Cs-methanesulfonate-based solution was used to investigate I-V relationships of evoked EPSCs, **Figure 6**).

Biocytin (0.2%) was routinely added to the patch solution for further cell reconstruction. Pyramidal neurons expressing GFP (green) were visually identified in acute slices (mPFC layer III-V) using fluorescence microscope. Electrophysiological recordings were made using a Multi Clamp 700A amplifier (Axon Instruments, Union City, CA, United States), operating under current-clamp and voltage-clamp mode. Data were filtered at 4 kHz and acquired using pClamp 10 software (Molecular devices, Sunnyvale, CA, United States). Local cortical inputs were electrically stimulated via a patch micropipette placed in the mPFC layer II. All recordings were done at a holding potential -70 mV. For the I-V curve experiments holding potential was varied from -100 mV to 60 mV. Paired-pulse stimulation was delivered with 50 ms interval. Action potentials (APs) were triggered using 500 ms depolarizing pulses of various amplitudes. The uncompensated series resistance was monitored by the delivery of -10 mV steps throughout the experiment, only recordings with less than 15% change were analyzed.

### Drugs

10 μM CNQX, 50 μM AP5 and 10 μM bicuculline methiodide (Sigma-Aldrich, Oakville, ON, Canada) were dissolved in extracellular ACSF and applied through the perfusion system (at least 5 min before recordings).

### Immunofluorescent Staining

Mice were euthanized 3 weeks after viral delivery by a lethal dose of ketamine/xylazine and perfused with phosphate buffer saline (PBS) followed by 4% paraformaldehyde (PFA). Brains were incubated in 4% PFA 24 h at 4°C. Fixed tissue was sectioned using vibratome (Leica, VT1000S). Next, 40 μm sections were boiled for 2 min in sodium citrate buffer (10 mM tri-sodium citrate dehydrate, 0.05% Tween-20, pH 6.0) and cooled down at room temperature (RT) for 20 min. Sections were blocked and permeabilized with a permeabilization solution containing 10% normal goat serum (NGS) and 0.5% Triton X-100 (Sigma) in PBS for 2 h. Sections were incubated with primary antibodies diluted in permeabilization solution overnight at 4°C. After three washes in PBS, samples were incubated with secondary antibodies for 2 h at room temperature. After washing with PBS three times, sections were mounted using DAKO mounting medium (DAKO, Mississauga, ON, Canada) and visualized with a confocal microscope (Zeiss LSM 700, Zen 2011 Software, Oberkochen, Germany). Quantification was performed using ImageJ (National Institute of Health (NIH), Bethesda, MD, United States).

For immunofluorescent staining of biocytin-filled neurons, acute brain slices were fixed in 4% PFA overnight at 4°C. Slices were washed 3 times in PBS and incubated in permeabilization solution containing 10% normal goat serum (NGS) and 0.5% Triton X-100 (Sigma-Aldrich, Oakville, ON, Canada) in PBS for 2 h at RT. After sections were incubated with streptavidin-Alexa 546 conjugated antibodies diluted in permeabilization solution overnight at 4°C. After washing with PBS three times, sections were mounted using the mounting medium (DAKO, Mississauga, ON, Canada). Pictures were taken using a confocal microscope (Zeiss LSM 700) with a voxel size of 0.1 × 0.1 × 0.55 μm. Spines counting and dendrite length measurements were performed blindly using NeuronStudio (Icahn School of Medicine at Mount Sinai (ISMMS, New York, NY, United States).

Following primary antibodies were used: mouse anti-Gsk3β (1:500, Abcam 93926, Cambridge, United Kingdom, for **Figure 1**), rabbit anti-Gsk3β (1:500, Cell Signal Technology 9315, Danvers, MA, United States, for Supplementary Figure S1) and Streptavidin-Alexa546 (1:200, Life Technologies/Thermo Fisher Scientific, S11225, Waltham, MA, United States) Secondary antibodies: Alexa Fluor 568 (Life Technologies/Thermo Fisher Scientific, Waltham, MA, United States, 1:1000).

### Tissue Dissection

Mice were killed by rapid cervical dislocation. Heads of animals were immediately cooled by immersion in liquid nitrogen for 6 s. mPFC tissues were dissected rapidly (within 30 s) on an ice-cold surface and frozen in liquid nitrogen. For synaptosome extraction experiments (**Figure 7**), first, 500 μm thick serial coronal sections were prepared using ice-cold adult mouse brain slicer and matrix (Zivic instruments), second, mPFC was dissected on ice cold surface using a microsurgical knife (KF Technology).

### Synaptosome Isolation and Western Blot

Synaptosomes were isolated using Syn-PER reagent according to manufacturer’s recommendations (Thermo Fisher Scientific). Briefly, dissected and frozen brain tissue was lysed in Syn-PER solution supplied with protease inhibitor cocktail, 10 mM NaFluoride, 25 mM βglycerophosphate, 10 mM Na Orthovanadate (Sigma-Aldrich, Oakville, ON, Canada). Samples were centrifuged for 10 min at 1200 *g*. After discarding the pellet, samples were centrifuged for another 20 min at 15000 *g* to obtain synaptosomes in the pellet. Neuro2A cells and dissected brain tissue were lysed in lysis buffer containing: 50 mM Tris-HCl, 150 mM NaCl, 5 mM EDTA, Protease inhibitor cocktail, 1% SDS, 0.5% Na-deoxycholate, 1% NP-40, 10 mM NaFluoride, 25 mM βglycerophosphate, 10 mM Na Orthovanadate (Sigma-Aldrich, Oakville, ON, Canada). Lysates were centrifuged 10000 *g* for 30 min and supernatants were collected. Protein concentration was measured by using a DC-protein assay (Bio-Rad, Hercules, CA, United States). Protein extracts were separated on precast 10% Tris-glycine gels (Thermo Fisher Scientific, Waltham, MA, United States) and transferred to nitrocellulose membranes. Blots were immunostained overnight at 4°C with primary antibodies. Immune complexes were revealed using appropriate IR dye-labeled secondary antibodies from Li-Cor Biotechnology (Lincoln, NE, United States). Quantitative analyses of fluorescent IR dye signal were carried out using Odyssey Imager and Image Studio Lite 5.2 software (Licor Biotechnology, Lincoln, NE, United States). For quantification, GAPDH (Actin in case of Neuro2A cells) was used as a loading control for the evaluation of total protein levels. For measurement of synaptic receptor levels, the ratio of p845GluA1/GluA1 and p880GluA2/GluA2 were calculated. Results were further normalized to respective control conditions to allow for comparison between separate experiments. Following primary antibodies were used in the experiments: mouse anti-Actin (1:10000, Millipore, MAB1501), mouse anti-GAPDH (1:5000, Santa Cruz sc-322333) rabbit anti-Gsk3β (1:500, Cell Signal Technology 9315, Danvers, MA, United States), rabbit anti-Fxr1 (1:1000, Abcam 129089), mouse anti-GFP (1:1000, Rockland/VWR 600-301-215), mouse anti-GluA1 (1:1000, Millipore MAB2263), rabbit anti-p845 (GluA1) (1:1000, Millipore 06-773), mouse anti-GluA2 (1:1000, Millipore MAB397), rabbit anti-p880 (GluA2) (1:1000, Abcam ab52180), mouse anti-NR1 N308/48 (1:1000, Antibodies incorporated 75-272), mouse anti-Vglut1 N28/9 (1:5, Antibodies incorporated 75-066), mouse anti-GABA_A_R alpha 1 N95/35 (1:1000, Antibodies incorporated 75-136), mouse anti-Neuroligin 1 (1:1000, Synaptic systems 129111), rabbit anti-Neuroligin 2 (1:1000, Synaptic systems 129202) and mouse anti-PSD95 (1:250, BD transduction 610495) Secondary antibodies: goat anti-mouse IR Dye 680 (1:10000, Mandel 926-68020), goat anti-rabbit IR Dye 800 (1:10000, Mandel 926-32211).

### Chemogenetic Inhibition

To activate KORD receptors and silence neurons, Salvinorin B (10 mg/kg) (SalB) (Cayman chemical, Ann Arbor, MI, United States) [or dimethyl sulfoxide (DMSO) as a vehicle] was administered to mice 4 weeks after stereotaxic injection of AAV9 KORD virus. SalB was dissolved in DMSO and injected subcutaneously (s.c.) at a volume of 1 μL/g body weight 10 min prior to behavioral test as described (Vardy et al., 2015).

### Behavioral Tests

#### Open field Test (OFT)

It was performed for 30 min in an automated Omnitech Digiscan apparatus (AccuScan Instrument, Columbus, OH, United States). Each mouse was placed in a corner of the large plexiglass box and the exploratory activity was recorded. Time spent in the center, number of entries and horizontal activity were recorded separately for the central (25% of the total surface) and peripheral areas.

#### Dark-Light Emergence Test (DLET)

It was performed for 5 min with mice placed initially at the center of the dark chamber. Tests were conducted using an automated open field activity apparatus with light/dark insert (Med-Associates, St Albans, VE, United States) with the light compartment illuminated at 800 lux. The total time spent in the dark and light compartments, the total distance traveled, and the delay to cross from the dark to the light chamber were used as parameters for analysis.

#### Elevated Plus Maze (EPM)

It was performed for 10 min with mice initially placed at the far end of the close arm. The time spent in the open arm was measured manually (by the observer being unaware of the treatment) and used for the analysis.

#### Behavioral *Z* Scoring

To obtain integrated measures in each group, emotionality- and locomotion-related data were normalized using a *Z*-score methodology (Guilloux et al., 2011). *Z*-scores for individual animals were calculated using the formula: Z = (X-μ)/σ, which indicates how many standard deviations (σ) an observation (X) is above or below the mean of a control group (μ). *Z*-scores for behavioral measures were first averaged within the test, and then across all three tests (OFT, DLET, EPM). OFT (time spent in the center), DLET (time spent in the light chamber), EPM (time in open arms) were used to obtain emotionality *Z*-scores. Locomotion *Z*-scores were obtained from DLET (total distance traveled) and OFT (total distance traveled) data.

### Quantification and Statistical Analysis

Synaptic events were analyzed using pClamp 10 software within at least 3 min of recordings, individual events were detected using automatic template search. Templates were created using the average of at least 10 events aligned by the rising of their slopes. The peak amplitude of evoked EPSCs (eEPSCs) was measured for an averaged response (5 trials). Paired-pulse ratio was calculated as average for 15–20 trials. Rectification index (RI) was calculated, as a ratio of *I*–*V* slopes, RI = *s*2/*s*1(Adesnik and Nicoll, 2007; Lalanne et al., 2016). First we calculated slope 1 (s1) using linear regression to *AMPA currents* recorded at holding potential ≤ 0 mV, as well as an AMPAR reversal potential, *E*_rev_. Next, we estimated slope 2 (s2) using linear fit of *I*–*V* data recorded at positive holding potentials and constrained to intersect the *x*-axis at *E*_rev_. This method allows taking into account variations of AMPA reversal potential between recordings. Threshold current necessary to evoke single AP, as well as maximal firing rate, AP amplitude, half width and time to peak (TTP) were calculated to investigate excitability.

The data are presented as means ± SEM. For comparison between two groups, two-tailed *t*-test is used. For comparison between multiple groups one-way ANOVA is used followed by Bonferroni-corrected pair-wise comparisons using GraphPadPrism 5 software (La Jolla, CA, United States) (^∗^*p* < 0.05, ^∗∗^*p* < 0.01, ^∗∗∗^*p* < 0.001).

## Results

### CRISPR/Cas9 Mediates Efficient Somatic Knockout of *Gsk3b*

Gsk3β activity or expression can be manipulated systemically by using various drugs or systemic genetic manipulations (Hoeflich et al., 2000; McManus et al., 2005; Beaulieu et al., 2009). Cell type or brain region specific inactivation of Gsk3β has also been achieved using the Cre-Lox system in transgenic mouse models (Latapy et al., 2012; Del’Guidice et al., 2015; Ochs et al., 2015). To avoid developmental compensation and preserve cell type and brain region specificity we took advantage of a non-conventional CRISPR/Cas9 method to induce sKO of *Gsk3b* in neurons of the adult mouse medial prefrontal cortex (mPFC, the mouse homolog of human DLPFC). First, we designed guide RNAs (gRNAs) targeting several exons of the *Gsk3b* gene using online CRISPR design tool to minimize off-target activity (**Figure 1A**). Efficacy of single-guide RNAs (sgRNAs) to target genomic DNA was tested *in vitro* by SURVEYOR assay following transfection into mouse cells (**Figure 1B**). Efficacy of the most active gRNA for *Gsk3b* (gRNA3) was further established, as compared to a scrambled gRNA, using a puromycin selection system (Ran et al., 2013) in mouse neuroblastoma cells. Expression of CRISPR/Cas9 against *Gsk3b* in transfected Neuro2A cells resulted in a massive decrease in expression levels of the Gsk3β protein. Moreover, KO of *Gsk3b* resulted in an increase of Fxr1 levels further validating the negative regulation of Fxr1 by Gsk3β (**Figures 1C,D**) (Del’Guidice et al., 2015).

A dual AAV viral delivery of CRISPR/Cas9 (Swiech et al., 2015) was used for *in vivo* applications. This system comprises one AAV vector encoding the Cas9 nuclease expressed under the neuron-specific short mecp2 promoter (AAV spCas9). The second AAV vector encodes *Gsk3b* targeting sgRNA3 expressed under a U6 promoter and a GFP-KASH fusion protein under the neuron-specific human synapsin (hSYN) promoter (AAV Gsk3 sgRNA/GFP) (Swiech et al., 2015). AAV SpCas9 and AAV Gsk3 sgRNA/GFP viral particles were mixed in 1:1 ratio and injected into the mouse medial prefrontal cortex (Gsk3 sKO condition). A mixture of AAV spCas9 and AAV GFP viral particles were used as a control (CRISPR-Ctrl condition) (**Figure 1E**). Mice were sacrificed 3 weeks after infection and Gsk3β expression was evaluated by immunofluorescent staining of brain slices using two different antibodies (**Figure 1F** and Supplementary Figure S1). Intense signal was detected throughout all brain slices since Gsk3β is ubiquitously expressed in neurons, astrocytes, and microglia (Perez-Costas et al., 2010). All GFP expressing control neurons (infected with AAV spCas9 + AAV GFP) showed expression of Gsk3β in their cell bodies. In contrast, 63% of GFP expressing Gsk3 sKO neurons (infected with AAV spCas9 + AAV Gsk3 sgRNA/GFP) had undetectable levels of Gsk3β (**Figures 1F,G**). Moreover, on the same brain slice absence of Gsk3β expression was only noted in virus infected neurons (infected with AAV spCas9 + AAV Gsk3sgRNA/GFP), while neurons outside of the infection area expressed Gsk3β (Supplementary Figure S1). Hence, *in vivo* delivery of CRISPR/Cas9 resulted in efficient, brain region targeted and neuron-specific sKO of *Gsk3b* gene.

To overexpress Fxr1 (Fxr1 over condition), we delivered a GFP-Fxr1 fusion construct to the mPFC using AAV (Del’Guidice et al., 2015). AAV GFP was used as a control (Ctrl condition) (**Figure 1E**). Mouse mPFCs were dissected and expression of Fxr1 was evaluated by western blot (**Figure 1H**). Expression of GFP-Fxr1 protein was detected by both anti-GFP and anti-Fxr1 antibodies in mice from the Fxr1 over condition, as opposed to Ctrl mice were only expression of brain-specific Fxr1 isoforms and GFP protein were detected (**Figure 1H**).

### Medial Prefrontal Cortex Specific Overexpression of Fxr1 or *Gsk3b* Somatic Knockout Result in Reduced Anxiety-Related Behaviors

**FIGURE 2:**
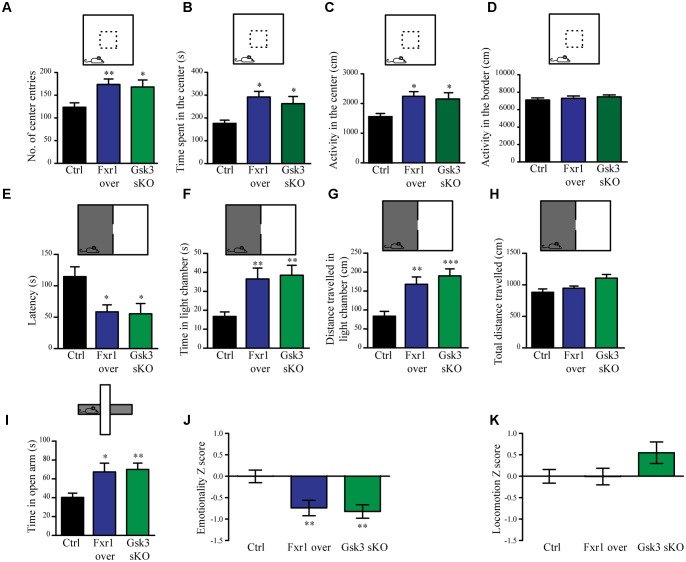
Prefrontal CRISPR/Cas9 mediated *Gsk3b* sKO or Fxr1 overexpression modulate mood related behaviors. **(A–D)** Open field test for control (*n* = 18), Fxr1 overexpressing (*n* = 16) and Gsk3 sKO (*n* = 17) mice. Graphs represent **(A)** number of center entries (Ctrl 123.6 ± 9.8, Fxr1 over 173.8 ± 11.9, and Gsk3 sKO 168.2 ± 15.3), **(B)** time spent in the center (Ctrl 176.4 s ± 14.2, Fxr1 over 292.3 s ± 24.3, Gsk3 sKO 262.8 s ± 31.5), **(C)** horizontal activity in the center (Ctrl 1553 cm ± 114, Fxr1 over 2244 cm ± 156, Gsk3 sKO 2154 cm ± 108) and **(D)** horizontal activity in the border (Ctrl 7106 ± 242 cm, Fxr1 over 7298 ± 267 cm and Gsk3 sKO 7468 ± 218). **(E–H)** Dark/light emergence test for control (*n* = 17), Fxr1 overexpressing (*n* = 11) and Gsk3 sKO (*n* = 10) mice. Graph represents **(E)** latency to cross from the dark to the light compartment (Ctrl 114.5 s ± 15.8, Fxr1 over 58.5 s ± 11.1, and Gsk3 sKO 55.4 s ± 11.4), **(F)** time spent in the light chamber (Ctrl 16.7 s ± 2.3, Fxr1 over 36.5 s ± 5.7, Gsk3 sKO 38.5 s ± 5.2), **(G)** distance traveled in the light chamber (Ctrl 83.5 cm ± 12.6, Fxr1 over 168.1 cm ± 19, Gsk3 sKO 190.2 cm ± 18.4) and **(H)** total distance traveled during all 5 min of the test (Ctrl 882.8 cm ± 52.3, Fxr1 over 947.1 cm ± 35.1, Gsk3 sKO 1107 cm ± 57.5). **(I)** Elevated plus maze test for control (40.3 s ± 4.5, *n* = 20), Fxr1 overexpressing (67.3 s ± 9.2, *n* = 16) and Gsk3 sKO (70 s ± 6.6, *n* = 20) mice. Graph represents time spent in open arms during all 10 min of the test. **(J)** Emotionality *Z*-score for control (–0.0001719 ± 0.1489, *n* = 21), Fxr1 overexpressing (–0.7383 ± 0.1835, *n* = 18) and Gsk3 sKO (–0.7108 ± 0.1589, *n* = 20) mice **(K)** Locomotion *Z*-score for control (3.6^∗^10^-6^± 0.157, *n* = 21), Fxr1 overexpressing (–0.004789 ± 0.196, *n* = 18) and Gsk3 sKO (0.545 ± 0.255, *n* = 20) mice. Error bars show standard error of the mean (SEM). (^∗^*p* < 0.05, ^∗∗^*p* < 0.01, ^∗∗∗^*p* < 0.001, one way ANOVA).

The interaction between functional polymorphisms of *GSK3B* and *FXR1* has been associated to the regulation of mood and emotionality in healthy subjects (Del’Guidice et al., 2015). Thus, we evaluated anxiety-related behavioral outcomes after augmentation of Fxr1 and reduction of Gsk3β levels. Mice were injected into mPFC with either: AAV GFP-Fxr1 (Fxr1 over condition), AAV GFP (Ctrl condition), AAV spCas9 + AAV Gsk3sgRNA/GFP (Gsk3 sKO condition) or AAVspCas9 + AAV GFP (CRISPR-Ctrl condition). Mice were subjected to behavioral tests 3 weeks after viral infection. Mice from the CRISPR-Ctrl and Ctrl condition did not show difference in behavioral tests, indicating that expression of Cas9 does not affect behavioral responses by itself in these tests (Supplementary Figures S2A–K). From this point on, Ctrl group consisted of an equal number of mice from CRISPR-Ctrl and Ctrl conditions. Fxr1 overexpression and sKO of *Gsk3b* in mPFC resulted in anxiolytic-like behaviors compared to controls in three separate behavioral paradigms: the open field exploration tests (**Figures 2A–D**), the dark light emergence tests (**Figures 2E–H**) and the elevated plus maze (**Figure 2I**). To obtain integrated measures for each group of mice and summarize results across all the tests, we performed behavioral *Z*-scoring (Guilloux et al., 2011). Mice from Fxr1 over and Gsk3 sKO groups showed a decrease in emotionality Z score compared to Control mice, while locomotion Z score was unaffected (**Figures 2J,K**). This indicates that either selective increase in the expression of Fxr1 or knockout of *Gsk3b* in mPFC neurons is sufficient to reduce anxiety.

### Prefrontal Overexpression of Fxr1 or *Gsk3b* sKO Reduce Excitatory Synaptic Currents

**FIGURE 3:**
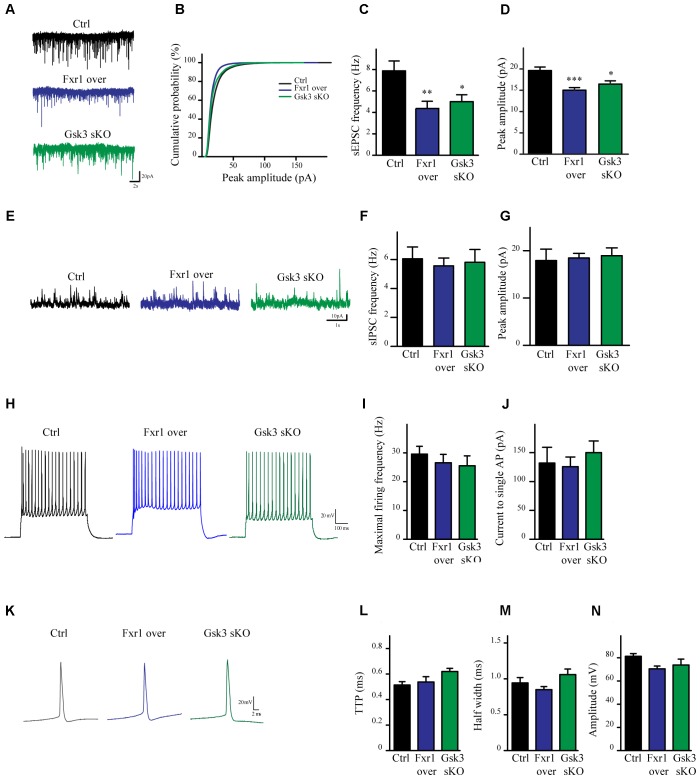
Prefrontal CRISPR/Cas9 mediated *Gsk3b* sKO or Fxr1 overexpression modulate the spontaneous neuronal activity. **(A)** Individual traces showing spontaneous EPSCs recorded from cortical slices of control, Fxr1 overexpressing and Gsk3 sKO mice. **(B)** Cumulative probability plot of sEPSCs amplitude (500 events per cell, control *n* = 26, Fxr1 overexpression *n* = 20, or Gsk3 sKO *n* = 19). sEPSCs **(C)** frequency and **(D)** peak amplitude in control (Frequency 7.87Hz ± 0.92, amplitude 19.61 pA ± 0.82), Fxr1 overexpressing (Frequency 4.36 Hz ± 0.67, amplitude 15.01 pA ± 0.62) and Gsk3 sKO (Frequency 4.99 Hz ± 0.65, amplitude 16.45 pA ± 0.75) neurons. **(E)** Individual traces showing spontaneous IPSCs recorded from cortical slices of control, Fxr1 overexpressing and Gsk3 sKO mice. Summary bar graphs showing **(F)** frequency and **(G)** peak amplitude of control (frequency 6.05Hz ± 0.81, amplitude 17.9 pA ± 2.41), Fxr1 overexpressing (frequency 5.56 Hz ± 0.54, amplitude 18.46 pA ± 0.96) and Gsk3 sKO neurons (frequency 5.80 Hz ± 0.88, amplitude 18.93 pA ± 1.65). **(H)** Individual traces showing trains of action potentials recorded from cortical slices of control, Fxr1 overexpressing and Gsk3 sKO mice. **(I,J)** Graphs showing excitability of control, Fxr1 overexpressing and Gsk3 sKO cells, **(I)** maximal firing rate (Ctrl 29.6 ± 3.5, Fxr1 over 26.5 ± 3.4, Gsk3 sKO 25.5 ± 3.5), **(J)** current necessary to evoke single action potential (Ctrl 132 ± 27, Fxr1 over 125 ± 16, Gsk3 sKO 150 ± 20). **(K)** Individual traces showing single action potential recorded from cortical slices of control, Fxr1 overexpressing and Gsk3 sKO mice. **(L–N)** Graphs showing action potential properties of control, Fxr1 overexpressing and Gsk3 sKO cells, **(L)** time to peak (TTP) (Ctrl 0.51 ± 0.02, Fxr1 over 0.53 ± 0.04, Gsk3 sKO 0.62 ± 0.02), **(M)** half-width (Ctrl 0.94 ± 0.07 Fxr1 over 0.85 ± 0.04 Gsk3 sKO 1.06 ± 0.07) and **(N)** amplitude (Ctrl 81.2 ± 2.3, Fxr1 over 70.5 ± 2.3, Gsk3 sKO 73.8 ± 4.25). Error bars show standard error of the mean (SEM). (^∗^*p* < 0.05, ^∗∗^*p* < 0.01, ^∗∗∗^*p* < 0.001, one way ANOVA).

To evaluate the impact of elevated Fxr1 and reduced Gsk3β levels on neuronal activity acute brain slices were obtained from mice and whole cell patch clamp recordings were performed on mPFC layer III-V pyramidal neurons. Fxr1 overexpression and *Gsk3b* sKO resulted in decreased spontaneous excitatory postsynaptic current (sEPSC) amplitude and frequency as compared to control (**Figures 3A–D**). In contrast, no changes of spontaneous inhibitory postsynaptic currents (sIPSCs) were detected (**Figures 3E–G**). Neuronal excitability (**Figures 3H–J**) and action potentials properties (**Figures 3K–N**) were unaffected by overexpression of Fxr1 and sKO of *Gsk3b*. Overall, this data indicates that augmentation of Fxr1 and reduction of Gsk3β levels has a major impact on spontaneous excitatory activity.

### KORD Mediated Silencing of mPFC Pyramidal Neurons Reduce Anxiety-Related Behaviors

**FIGURE 4:**
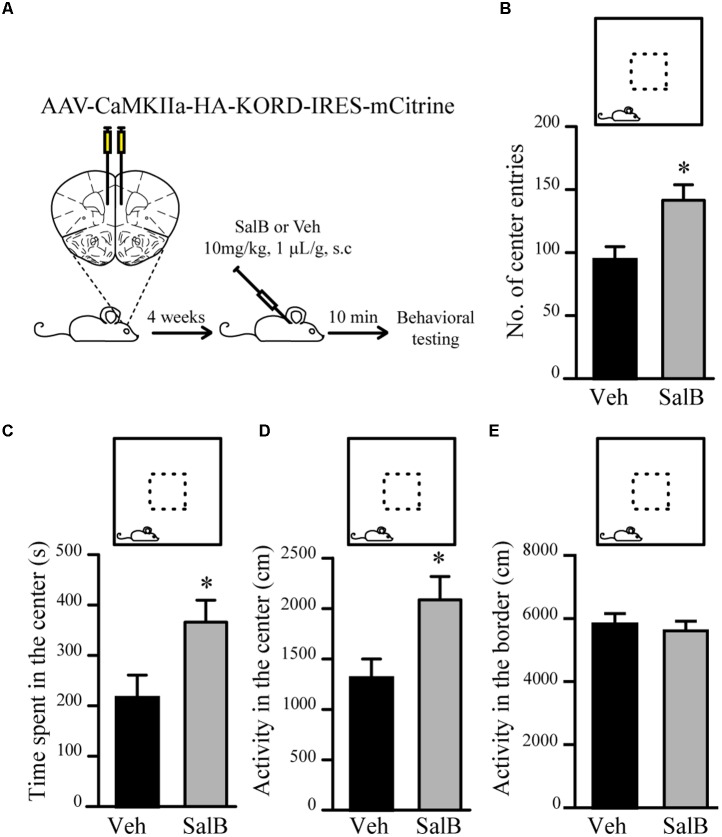
KORD mediated silencing of mPFC pyramidal neurons modulate anxiety related behavior. **(A)** Schematic diagram of experimental design. **(B–E)** Open field test for KORD-injected mice receiving vehicle (veh) (*n* = 8) or salvinorinB (SalB) (*n* = 8). **(B)** Number of center entries (veh 94.4 ± 10.4, SalB 141.6 ± 12.3), **(C)** time spent in the center (veh 216.2 s ± 44.6, SalB 366.2 s ± 43.5), **(D)** horizontal activity in the center (veh 1310 cm ± 189, SalB 2087 cm ± 230) and **(E)** horizontal activity in the border (veh 5818 cm ± 313, SalB 5609 cm ± 287). Error bars show standard error of the mean (SEM). (^∗^*p* < 0.05, ^∗∗^*p* < 0.01, ^∗∗∗^*p* < 0.001, Student’s *t*-test).

To verify if reduced excitatory neuronal activity in pyramidal neurons of the mPFC can be associated to a reduction in anxiety-related behaviors, we used κ-opioid derived DREADD (KORD) (Vardy et al., 2015) mediated silencing of mPFC neurons. KORD is an engineered Gαi protein-coupled kappa opioid receptor that can be specifically activated by the biologically inert drug salvinorin B (SalB) leading to neuronal silencing (Vardy et al., 2015). One limitation of the DREADD technology is that CNO, the activator of muscarinic receptor derived DREADDs, is metabolized to Clozapine *in vivo* thus leading to potential side effects other than activation of DREADDs (Gomez et al., 2017; Mahler and Aston-Jones, 2018). The use of KORD allows to circumvent this limitation since SalB has no biological activity *in vivo* (Vardy et al., 2015). Since inhibitory neurotransmission is not affected by manipulations of Gsk3β and Fxr1 expression (**Figures 3E–G**), we sought to silence only excitatory neurons. To achieve this, an AAV vector with a CamKIIa promoter was used to express KORD only in pyramidal neurons of the mPFC (Wang et al., 2013). Four weeks after AAV KORD injection, mice were subjected to behavioral testing. One group of mice received vehicle (veh) and a second SalB (**Figure 4A**). Silencing of mPFC pyramidal neurons in response to the activation of KORD by SalB resulted in anxiolytic-like behaviors similar to those observed in mice from the Fxr1 over and Gsk3 sKO conditions (**Figures 4B–E**) thus establishing a functional association between reduced excitatory neuronal activity and behavioral outcomes.

### Prefrontal Overexpression of Fxr1 and *Gsk3b* sKO Does Not Affect Spine Density

**FIGURE 5:**
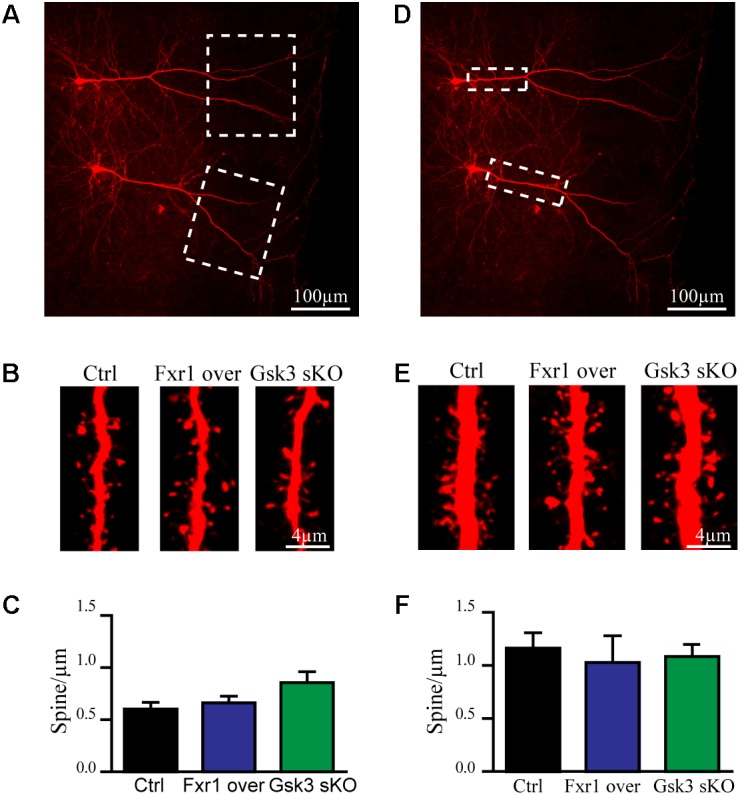
Prefrontal CRISPR/Cas9 mediated *Gsk3b* sKO or Fxr1 overexpression have no effect on spine density. **(A)** Representative picture of biocytin filled pyramidal neurons (dotted box represents the area of spine density quantification). **(B)** Representative pictures of spines on the distal part of the apical dendrite of control, Fxr1 overexpressing and Gsk3 sKO cortical neurons. **(C)** Quantification of spine density on the distal part of the apical dendrite of control (0.6 ± 0.065, *n* = 8 cells, 4 mice, 3270 spines), Fxr1 overexpressing (0.66 ± 0.064, *n* = 6 cells, 4 mice, 2124 spines) and Gsk3 sKO (0.85 ± 0.11, *n* = 6 cells, 4mice, 1747 spines) cortical neurons. **(D)** Representative picture of biocytin filled pyramidal neurons (dotted box represents the area of spine density quantification). **(E)** Representative pictures of spines on the proximal part of the apical dendrite of control, Fxr1 overexpressing and Gsk3 sKO cortical neurons. **(F)** Quantification of spine density on the proximal part of the apical dendrite of control (1.16 ± 0.14, *n* = 6 cells, 3 mice, 1223 spines), Fxr1 overexpressing (1.02 ± 0.25, *n* = 3 cells, 3 mice, 504 spines) and Gsk3 sKO (1.08 ± 0.14, *n* = 5 cells, 4 mice, 940 spines) cortical neurons. Error bars show standard error of the mean (SEM). (^∗^*p* < 0.05, ^∗∗^*p* < 0.01, ^∗∗∗^*p* < 0.001, one-way ANOVA).

Excitatory synapses are mostly localized in dendritic spines of pyramidal neurons (Peters, 2007). Furthermore, congenital reductions in expression of Fragile X family proteins have been shown to results in alterations of synaptic spine density (Comery et al., 1997; Cook et al., 2014; Guo et al., 2015). We have performed morphological analysis to address whether observed reduction in the frequency of EPSCs was a result of decreased spine density. Since spine density on apical dendrite of pyramidal neurons may vary, we subdivided apical dendrite into distal and proximal parts to minimize variability (**Figures 5A,D** dotted squares). No differences were found on distal (**Figures 5A–C**) or proximal (**Figures 5D–F**) apical dendrite spine density between Fxr1 over, Gsk3 sKO and Ctrl conditions. This indicates that changes in Fxr1 or Gsk3β expression levels do not induce major morphological alterations in synaptic spines of pyramidal neurons.

### Prefrontal Overexpression of Fxr1 or *Gsk3b* sKO Alters AMPA Receptor Mediated Currents

**FIGURE 6:**
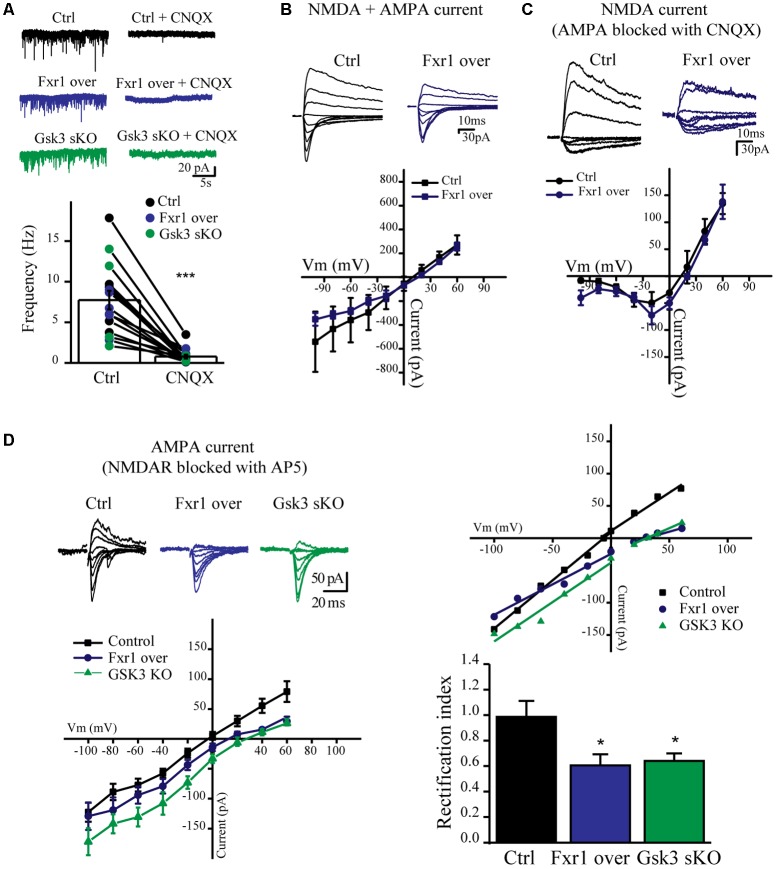
Prefrontal CRISPR/Cas9 mediated *Gsk3b* sKO or Fxr1 overexpression affect AMPA current. **(A)** Frequency of sEPSCs recorded from cortical slices in control (7.74Hz ± 1.18, *n* = 15) and after application of CNQX (0.78Hz ± 0.24, *n* = 15) (^∗^*p* < 0.05, ^∗∗^*p* < 0.01, ^∗∗∗^*p* < 0.001, paired Student’s *t*-test). **(B–C)** I-V curves of evoked EPSC amplitude recorded in control (black, *n* = 3) and Fxr1 overexpressing (blue, *n* = 3) brain slices **(B)** before and **(C)** after application of CNQX in the presence of bicuculline. Representative examples of recordings are shown as inserts on top. **(D)** Top Left: Representative traces of evoked EPSC amplitude recorded in control (black), Fxr1 overexpressing (blue) and Gsk3 sKO (green) slices in the presence of bicuculline and AP-5. Average graph (Bottom Left) and representative examples (Top Right) of I-V curves of evoked EPSC amplitude recorded in control (black), Fxr1 overexpressing (blue) and Gsk3 sKO (green) neurons from brain slices (Top Right: lines show linear fit to the data and calculated reversal potential is also included among data points). Bottom Right: summary bar graphs showing rectification index of control (black, 0.99 ± 0.13, *n* = 8), Fxr1 overexpressing (blue, 0.51 ± 0.08, *n* = 8) and Gsk3 sKO (green, 0.64 ± 0.06, *n* = 9) neurons. (^∗^*p* < 0.05, ^∗∗^*p* < 0.01, ^∗∗∗^*p* < 0.001, one-way ANOVA). Error bars show standard error of the mean (SEM).

We have observed a reduction of sEPSC amplitude in Fxr1 over and Gsk3 sKO conditions. Ionotropic AMPA and NMDA receptors are major players in mediating excitatory neurotransmission. Thus, we performed recordings in the presence of channel blockers to identify the main source of reduced excitatory currents. Bath application of AMPA receptor blocker CNQX drastically reduced amplitude and almost completely abolished frequency of recorded sEPSCs from all conditions (**Figure 6A**), indicating that recorded sEPSCs were mainly mediated by AMPA receptors. AMPA receptors are heterotetramers composed of a combination of four subunits (GluA1-4) (Hollmann and Heinemann, 1994). GluA1/GluA2 heterotetramers are the predominant AMPA receptors in the adult forebrain (Craig et al., 1993). GluA1 homotetramer AMPA receptors are Ca^+2^ permeable and inwardly rectifying (Jonas and Burnashev, 1995), thus changes in rectification index may indicate changes of AMPA receptor subunit composition. In order to calculate rectification index, we conducted current-voltage relationship (I-V curve) experiments using electric stimulation to evoke EPSCs. Overexpression of Fxr1 had no effect on I-V curve of evoked mixed AMPA + NMDA currents (**Figure 6B**) and isolated NMDA currents, recorded in the presence of CNQX (**Figure 6C**). In contrast, recordings of AMPA mediated EPSCs revealed a decrease of the rectification index in Fxr1 over and Gsk3 sKO conditions (**Figure 6D**), thus suggesting a change in the AMPA receptor composition corresponding to the prevalence of GluA1 homotetramer mediated currents.

### Prefrontal Overexpression of Fxr1 or *Gsk3b* sKO Affect Components of the Glutamatergic Synapse

**FIGURE 7:**
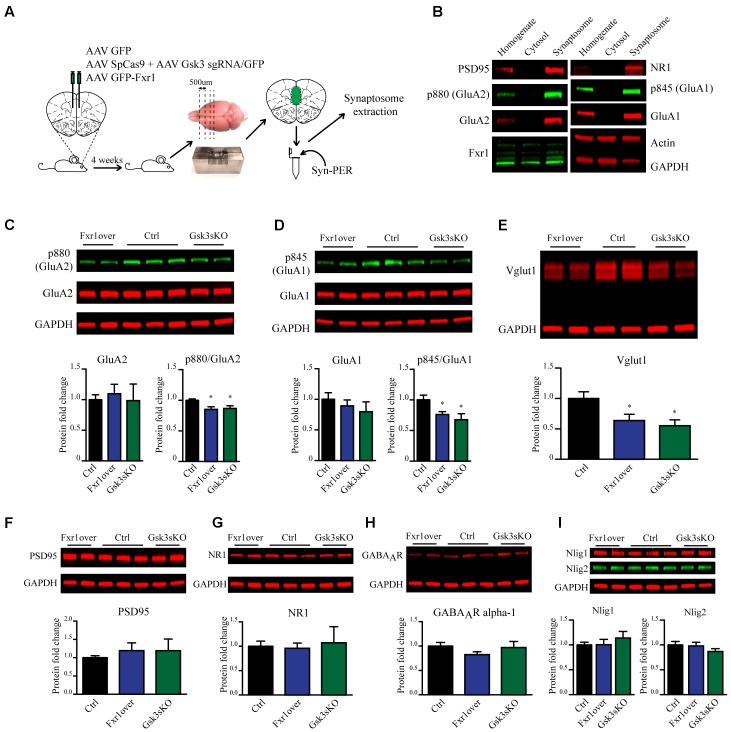
Prefrontal CRISPR/Cas9 mediated *Gsk3b* sKO or Fxr1 overexpression affect pre- and postsynaptic components of the glutamatergic synapse. **(A)** Schematic diagram of experimental design. **(B)** Visualization of candidate proteins by Western blot in Homogenate, Cytosol and Synaptosomal fractions. **(C)** Expression of AMPA receptor subunit GluA2 and its p880 phosphorylated form in synaptosomes from Ctrl (GluA2/GAPDH 1 ± 0.079, p880/GluA2 1 ± 0.021, *n* = 6 mice), Fxr1 overexpressing (GluA2/GAPDH 1.09 ± 0.15, p880/GluA2 0.85 ± 0.04, *n* = 7 mice) and Gsk3 sKO (GluA2/GAPDH 0.98 ± 0.27, p880/GluA2 0.86 ± 0.04, *n* = 5 mice) mice. A representative picture is shown on the top panel. **(D)** Expression of AMPA receptor subunit GluA1 and its p845 phosphorylated form in synaptosomes from Ctrl (GluA1/GAPDH 1 ± 0.05, p845/GluA1 1 ± 0.07, *n* = 6 mice), Fxr1 overexpressing (GluA1/GAPDH 1.17 ± 0.09, p845/GluA1 0.76 ± 0.04, *n* = 7 mice) and Gsk3 sKO (GluA1/GAPDH 1.16 ± 0.09, p845/GluA1 0.67 ± 0.09, *n* = 5 mice) mice. A representative picture is shown on the top panel. **(E)** Expression of Vglut1 in synaptosomes from Ctrl (Vglut1/GAPDH 1 ± 0.11, *n* = 6 mice), Fxr1 overexpressing (Vglut1/GAPDH 0.63 ± 0.1, *n* = 7 mice) and Gsk3 sKO (Vglut1/GAPDH 0.55 ± 0.09, *n* = 4 mice) mice. A representative picture is shown on the top panel. **(F)** Expression of PSD95 in synaptosomes from Ctrl (PSD95/GAPDH 1 ± 0.05, *n* = 6 mice), Fxr1 overexpressing (PSD95/GAPDH 1.19 ± 0.2, *n* = 7 mice) and Gsk3 sKO (PSD95/GAPDH 1.18 ± 0.03, *n* = 5 mice) mice. A representative picture is shown on the top panel. **(G)** Expression of NMDA receptor subunit NR1 in synaptosomes from Ctrl (NR1/GAPDH 1 ± 0.1, *n* = 6 mice), Fxr1 overexpressing (NR1/GAPDH 0.95 ± 0.1, *n* = 7 mice) and Gsk3 sKO (NR1/GAPDH 1 ± 0.3, *n* = 5 mice) mice. A representative picture is shown on the top panel. **(H)** Expression of GABA A receptor subunit alpha 1 in synaptosomes from Ctrl (GABA_A_R/GAPDH 1 ± 0.07, *n* = 6 mice), Fxr1 overexpressing (GABA_A_R /GAPDH 0.8 ± 0.05, *n* = 7 mice) and Gsk3 sKO (GABA_A_R /GAPDH 0.96 ± 0.12, *n* = 5 mice) mice. A representative picture is shown on the top panel. **(I)** Expression of Neuroligin 1 and Neuroligin 2 in synaptosomes from Ctrl (Nlig1/GAPDH 1 ± 0.04, Nlig2/GAPDH 2 1 ± 0.05 *n* = 6 mice), Fxr1 overexpressing (Nlig1/GAPDH 1 ± 0.1, Nlig2/GAPDH 0.97 ± 0.07, *n* = 7 mice) and Gsk3 sKO (Nlig1/GAPDH 1.13 ± 0.12, Nlig2/GAPDH 0.86 ± 0.05, *n* = 5 mice) mice. A representative picture is shown on the top panel. Error bars show standard error of the mean (SEM). (^∗^*p* < 0.05, ^∗∗^*p* < 0.01, ^∗∗∗^*p* < 0.001, Student’s *t*-test).

Fxr1 overexpression and *Gsk3b* sKO resulted in augmentation of GluA1 homomer mediated currents, while overall AMPA receptor-mediated sEPSCs were decreased. These changes can originate either from altered local translation in spines or from changes in trafficking and subsequent insertion of GluA1 and GluA2 subunits into the glutamatergic synapse. To address those questions we investigated the impact of Fxr1 overexpression and *Gsk3b* sKO directly on the level of GluA1 and GluA2 subunits. To be more selective for local protein expression in spines and enrich samples for synaptic proteins, we performed crude synaptosome isolation from dissected brain tissue (**Figure 7A**). As a validation, we found enrichment of synaptic proteins in our synaptosomal preparation (**Figure 7B**). Overexpression of Fxr1 and sKO of *Gsk3b* did not result in changes in the levels of GluA1 and GluA2 subunits in the synaptosomal preparation (**Figures 7C,D**). This indicates that local expression of AMPA receptor subunits may not be altered in these conditions. It has been shown that phosphorylation of GluA1 and GluA2 may be involved in regulation of their trafficking and anchoring to postsynaptic density, hence in their surface expression (Banke et al., 2000; Chung et al., 2000; Ehlers, 2000; Esteban et al., 2003; Seidenman et al., 2003; Steinberg et al., 2006; Man et al., 2007; Diering et al., 2014). Levels of both p845 GluA1 and p880 GluA2 were reduced in synaptosomes following *Gsk3b* sKO or Fxr1 overexpression (**Figures 7C,D**). This shows that synaptic AMPA receptor composition changes in these conditions are likely not due to changes in local synthesis, but rather altered trafficking of GluA1 and GluA2 subunits.

In addition to changes in synaptic AMPA receptor subunit trafficking, we identified a decrease in vesicular glutamate transporter Vglut1 indicating possible presynaptic alterations under Fxr1 over and Gsk3 sKO conditions (**Figure 7E**). No changes in expression level of synaptosomal PSD95 (**Figure 7F**), NMDA receptor subunit 1 (**Figure 7G**), GABA A receptor subunit alpha 1 (**Figure 7H**), Neuroligin1 and Neuroligin 2 (**Figure 7I**) were observed between Fxr1 over, Gsk3 sKO and Control conditions. Overall, augmentation of Fxr1 and reduction of Gsk3β in mPFC decrease both p845 GluA1 and p880 GluA2 subunits, as well as synaptosomal Vglut1. This indicates a broad impact of Fxr1-Gsk3β signaling on glutamatergic neurotransmission potentially affecting both pre- and post-synaptic compartments.

## Discussion

*FXR1* recently has been identified as a risk factor for schizophrenia and bipolar disorder (Consortium, 2014; Hauberg et al., 2016; Liu et al., 2016; Takata et al., 2017). Interaction between polymorphisms affecting cortical expression of the *FXR1* and *GSK3B* genes have been shown to regulate mood-related behavioral dimensions in healthy humans and patients with bipolar disorder (Del’Guidice et al., 2015; Bureau et al., 2017). This genetic interaction may be explained by the negative regulation of Fxr1 following its phosphorylation by Gsk3β (Del’Guidice et al., 2015; Qie et al., 2017). Results presented here demonstrate how neuronal activity and related behavior are impacted by Gsk3β and Fxr1. Mice with reduced Gsk3β or elevated Fxr1 expression in mPFC showed decreased anxiety-related behaviors and reduced AMPA mediated excitatory postsynaptic currents. Our results indicate that these effects originate from the capacity for the Gsk3β and Fxr1 to alter AMPA mediated glutamatergic neurotransmission by affecting synaptic GluA1 and GluA2 subunits as well as vesicular glutamate transporter Vglut1.

Systemic inhibition of Gsk3β activity has been shown to result in anti-depressant and anxiolytic-like behavioral effects in mice (Kaidanovich-Beilin et al., 2004; Beaulieu et al., 2008a,b, 2009). Similar behavioral signatures were reported using a conventional Cre-lox system to suppress Gsk3β expression in all CamKII expressing forebrain pyramidal neurons (Latapy et al., 2012) or in all the cells of the prefrontal cortex (Del’Guidice et al., 2015). Overexpression of Fxr1 in mPFC has been shown to have anxiolytic-like effect in DLET (Del’Guidice et al., 2015). Here we used CRISPR/Cas9 mediated sKO to achieve brain region targeted and neuron-specific modulation of *Gsk3b* gene expression in adult mice. Moreover, we expended the characterization of anxiety-related behaviors and obtained an integrated index from all the tests. Reduction of Gsk3β or elevation of Fxr1 levels in mPFC neurons resulted in similar anxiolytic-like behaviors, further validating *in vivo* relationship between these two proteins. The modulation of mood-related behaviors by Fxr1 and Gsk3β is in line with observations in human subjects carrying functional polymorphisms for *FXR1* and *GSK3B* genes (Del’Guidice et al., 2015). Interestingly, anxiety symptoms are highly comorbid in schizophrenia patients and *FXR1* being a risk factor for schizophrenia can represent a potential molecular target to study mood related problems in these patients (Braga et al., 2013; Temmingh and Stein, 2015). Overall, our results illustrate that alteration of Gsk3β and Fxr1 expression levels in mPFC neurons of adult mice is sufficient to modulate mood-related behaviors.

A reduction in the frequency and amplitude of sEPSC has been reported following the Cre/Lox mediated suppression of Gsk3β expression in CA1 pyramidal neurons in adult mice (Ochs et al., 2015). These effects have been suggested to result from increased beta-catenin levels. Here we show that modulation not only of Gsk3β but also its substrate Fxr1 in mPFC can result in similar electrophysiological outcomes with behavioral consequences. This shows the need to expand studies of Gsk3β targets and their involvement in the various functions of this kinase. This may lead to the identification of converging or diverging functional pathways involving different Gsk3β targets.

The modulation of neuronal activity by Gsk3β and Fxr1 in the mPFC is most probably linked to their effects on anxiety-related behaviors. Indeed, chemo-genetic KORD mediated silencing of mPFC pyramidal neurons caused anxiolytic-like responses, therefore supporting a link between neuronal activity and behavior. Interestingly, inhibition of the direct excitatory input from ventral hippocampus (vHPC) to mPFC has been shown to decrease anxiety in the elevated plus maze and open field test (Padilla-Coreano et al., 2016). The decrease of mPFC neuronal activity has also been associated with resilience in the learned helplessness model of depression (Wang et al., 2014). In contrast, increase of mPFC activity using chemogenetics has been reported to trigger helplessness in resilient mice in this same model (Wang et al., 2014). Along with our observations, this supports a role for the decreased neuronal activity of mPFC neurons in maintaining low emotionality and greater mood stability.

Recordings from brain slices showed that reduction of Gsk3β or augmentation of Fxr1 expression affects excitatory postsynaptic activity through modulation of AMPA receptors, which includes not only decrease in the sEPSC amplitude, but also change in the rectification index. Further investigation revealed that augmentation of Fxr1 and reduction of Gsk3β levels resulted in a decrease of both synaptic GluA1 and GluA2 subunits. These results indicate an overall decrease in synaptic AMPA receptors along with a possible switch from predominantly heteromeric GluA1/GluA2 containing to homomeric GluA1 AMPA receptors with higher rectification properties.

Apart from the autosomal Fxr1, the fragile X gene family comprises two other members Fmr1, which is encoded on the X chromosome and Fxr2, which is also autosomal. These proteins show strong structural homology but do not have fully overlapping functions (Siomi et al., 1995; Kirkpatrick et al., 2001; Bontekoe et al., 2002; Spencer et al., 2006; Zhang et al., 2009; Xu et al., 2011; Guo et al., 2015). In line with this, it has been shown that Fmr1, and Fxr2, are involved in the regulation of AMPA receptor subunits via distinct mechanisms (Guo et al., 2015). Fxr2 directly binds to the coding sequence of GluA1 and regulates its expression by stabilizing its mRNA, while Fmr1 only regulates surface levels of this AMPA receptor subunit with no effect on its expression levels (Guo et al., 2015). Regulation of GluA2 by Fxr2 has also been reported albeit with variable results (Cook et al., 2014; Guo et al., 2015). In hippocampal slices, Fxr1 has been reported to negatively regulate the *de novo* synthesis of the GluA2 subunit of AMPA receptors by directly binding to the 5’UTR of its mRNA, during chemically induced long-term potentiation. However, possible alterations of GluA1 subunit were not thoroughly investigated (Cook et al., 2014). Our results suggest a regulation of both AMPA receptor subunits by Fxr1. Interestingly Fxr1 and Gsk3β altered only p845 GluA1 and p880 GluA2 with no apparent changes in total synaptosomal expression levels of these AMPA receptor subunits. This could be indicative of a regulation on the level of receptor trafficking, however, the exact mechanism by which Fxr1 may regulate GluA1 and GluA2 subunits in this system remains to be investigated.

Changes in Fxr1 and Gsk3β levels did not result in alterations of synaptosomal PSD95, NMDA receptor subunit 1, GABA A receptor alpha 1, Neuroligin 1 and 2. This is in line with the absence of alterations in spine density as well as NMDA receptor and GABA receptor-mediated currents observed under the Fxr1 over and Gsk3 sKO conditions. However, those conditions resulted in a decrease of Vglut1 indicating possible alterations in presynaptic glutamate release. The decrease in Vglut1 has been shown to affect the quantal size and result in a reduction of frequency and amplitude of EPSCs (Wojcik et al., 2004). Thus, reduction in Vglut1, along with a reduction in GluA1 and GluA2, may contribute to decrease in amplitude and explain the decrease in the frequency of spontaneous EPSCs found in Fxr1 over and Gsk3 sKO conditions. Overall, this indicates that alteration in Fxr1 and Gsk3β expression may have both pre- and post-synaptic impact on spontaneous glutamatergic neurotransmission.

## Conclusion

Our results showcase that a disease-associated factor Fxr1 and its regulator Gsk3β modulate components of neuronal signaling and impact behavioral manifestations in the same manner. Inhibition of GSK3 activity has been suspected for a long time to contribute to the behavioral actions of psychoactive drugs such as lithium, antidepressants, and antipsychotics (Beaulieu et al., 2009; Beurel et al., 2011). The correlation between the effects of Gsk3β inactivation and Fxr1 overexpression suggests that this RNA binding protein may be one of the major substrate through which Gsk3β exerts these effects by modulating glutamatergic synapses. Further manipulation of Gsk3β-Fxr1 signaling in different brain regions and cell types may allow uncovering the molecular and circuit level underpinnings of various phenotypes impacted by this signaling. This, in turn, could shed light on the pathophysiology of mental disorders and lead to the rational development of novel therapeutics.

## Author Contributions

J-MB and JK conceived the study and designed the experiments. J-MB, JK, and AE wrote the manuscript. JK performed the design and testing of CRISPR/Cas9 *in vitro* and *in vivo*, stereotaxic injections, brain dissections and synaptosome preparations, protein expression analysis *in vitro* and *in vivo*, mouse behaviors, spine counting, and data analysis. AE and SC performed whole cell patch clamp recordings and data analysis. AM performed CRISPR/Cas9 KO experiments with puromycin selection followed by detection of Gsk3β and Fxr1 expression. AB performed behaviors of KORD-injected mice. KT provided technical, financial, and intellectual support.

## Conflict of Interest Statement

The authors declare that the research was conducted in the absence of any commercial or financial relationships that could be construed as a potential conflict of interest.

## Footnotes

1 http://crispr.mit.edu/
