# Supplementary material for: Mental Illnesses-Associated Fxr1 and Its Negative Regulator Gsk3β Are Modulators of Anxiety and Glutamatergic Neurotransmission
Source: Front Mol Neurosci. 2018 Apr 12;11:119. doi: 10.3389/fnmol.2018.00119 (PMC5906571; doi:10.3389/fnmol.2018.00119)
Supplement: Supplementary file 1 [file Image_1.PDF]

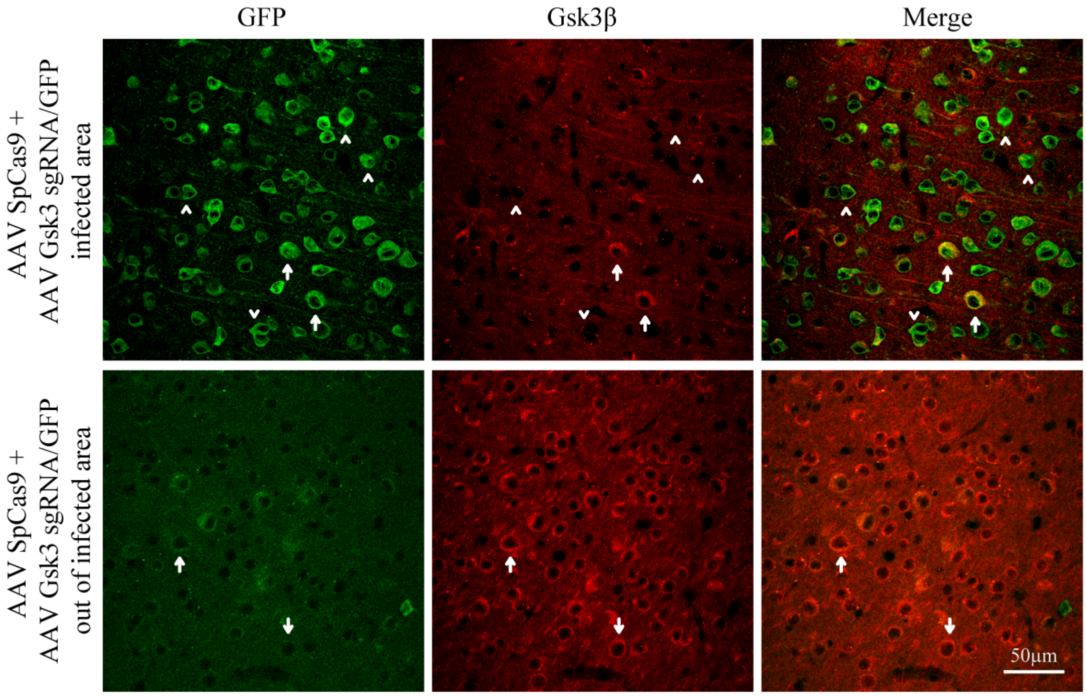

Figure S1

Supplementary Figure S1.

Immunofluorescent staining of virus injected brain sections with Gsk3β antibody (Gsk3β red, GFP green). Arrows indicate GFP+Gsk3β+ (double positive) cells, arrowheads indicate cells only positive for GFP. Note absence of Gsk3β+ cells only in virus infected area in contrast to out of virus infected area.

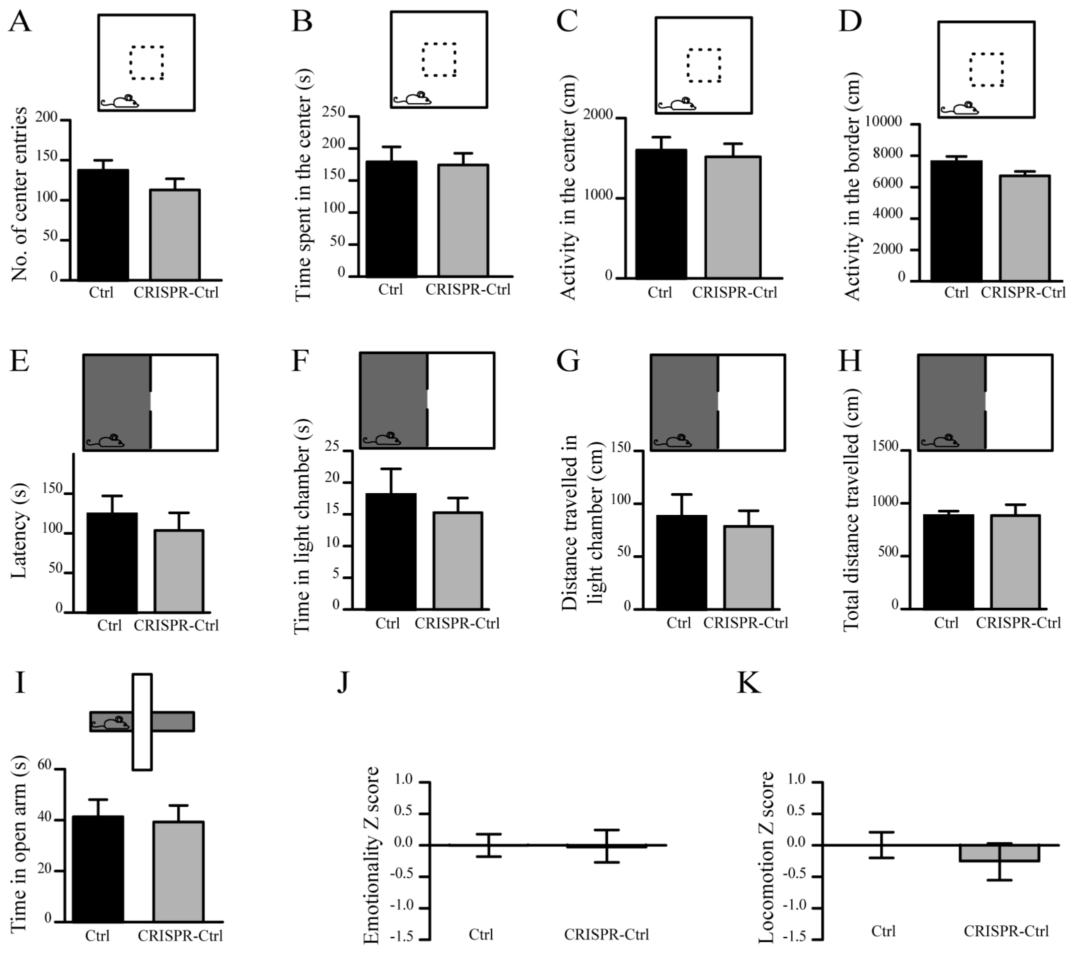

Figure S2

Supplementary Figure S2. Cas9 expression does not alter anxiety-related behaviors

(**A-D**). Open field test for mice injected with AAV GFP (n=8) and AAV SpCas9 + AAV GFP (n=10). Graphs represent (**A**) Center entries (AAV GFP 137±12.8, AAV SpCas9 + AAV GFP 112.8±14), (**B**) time spent in the center (AAV GFP 178.9s±23.7, AAV SpCas9 + AAV GFP 174.3s±18.2), (**C**) horizontal activity in the center (AAV GFP 1597cm±165, AAV SpCas9 + AAV GFP 1518±163) and (**D**) horizontal activity in the border (AAV GFP , AAV SpCas9 + AAV GFP). (**E-H**). Dark-light emergence test for mice injected with AAV GFP (n=9) and AAV SpCas9 + AAV GFP (n=8). Graphs represent (**E**) Latency to cross the border from dark to light

compartment (AAV GFP 124.3s±23, AAV SpCas9 + AAV GFP 103.5s±22.3), (**F**) time in the light chamber (AAV GFP 18.1s±4, AAV SpCas9 + AAV GFP 15.2s±2.3), (**G**) distance travelled in the light chamber (AAV GFP 88.1cm±20.6, AAV SpCas9 + AAV GFP 78.5cm±14.9) and (**H**) total distance travelled during all 5 minutes of the test (AAV GFP 881.3cm±45.5, AAV SpCas9 + AAV GFP 884.4cm±103.2). (**I**). Elevated plus maze for mice injected with AAV GFP (41.3s±6.75 n=10) and AAV SpCas9 + AAV GFP (39.3s±6.45 n=10). Graph represents time spent in the open arm during all 10 minutes of the test. (**J**) Emotionality Z-score for mice injected with AAV GFP ($-5.47*10^{-5}$ ±0.1784, n=11) and AAV SpCas9 + AAV GFP (-0.02619±0.2433, n=10). (**K**) Locomotion Z-score for mice injected with AAV GFP ($9.021*10^{-6}$ ±0.6949, n=11) and AAV SpCas9 + AAV GFP (-0.2469±0.9687, n=10). Error bars show standard error of the mean (SEM). (*$p < 0.05$, **$p < 0.01$, ***$p < 0.001$, Student's *t*-test).
